# Adrenocortical Carcinoma: A Case Report on the Diagnostic and Therapeutic Approach

**DOI:** 10.7759/cureus.117195

**Published:** 2026-09-30

**Authors:** Efthymios Poulios, Sofia Theofilopoulou, Theodoros Sidiropoulos, Dimitrios Schizas, Spyridon Christodoulou

**Affiliations:** 1 4th Department of Surgery, Attikon University Hospital, University of Athens, Athens, GRC; 2 Medicine, Attikon University Hospital, National and Kapodistrian University of Athens, Athens, GRC; 3 1st Department of Surgery, National and Kapodistrian University of Athens, Laikon General Hospital, Athens, GRC

**Keywords:** adrenal cancer, adrenal lesions, adrenocortical carcinoma (acc), non-functioning adrenocortical cancer, retroperitoneal adrenalectomy

## Abstract

Although the diagnosis of adrenocortical carcinoma might not be challenging, there is still a lack of evidence on the recommended therapeutic options, the extent of lymph node dissection, and the use of chemotherapy and radiotherapy.

This is the case of a 43-year-old man who presented to our department with unregulated blood pressure. During the diagnostic workup, he underwent a CT scan that showed an incidental solid, ovoid 7-cm lesion of the adrenal gland with a density of 30 HU pre-contrast, 60 HU right after, and 38 HU in a 15-min delayed measurement after contrast administration. His plasma metanephrines, dehydroepiandrosterone sulfate (DHEA-S), total and free testosterone, and adrenocorticotropic hormone (ACTH) were normal. While his 8.00 am baseline serum cortisol was also within the age-adjusted reference range, the 8.00 am serum cortisol concentration after an overnight 1-mg dexamethasone suppression test was suppressed. Although the aldosterone-to-renin ratio was found to be elevated, this was attributed to concomitant β-adrenergic blockade due to the biochemical evaluation. The patient underwent an endoscopic retroperitoneal left adrenalectomy. Histological examination revealed an encapsulated adrenocortical carcinoma, with a Ki-67 proliferation index of 21%. The patient was discharged and remains under follow-up with no evidence of recurrence.

Adrenocortical carcinomas are classified into stages according to their size, local invasion, and metastatic potential. Surgical excision is the treatment of choice for non-metastasized ACCs, while in cases of metastases, chemotherapy and radiotherapy is usually preferred.

This case highlights the need for further research into the pathophysiology of this malignancy to facilitate the development of evidence-based guidelines and to optimize management strategies for advanced-stage disease.

## Introduction

Adrenal tumors are a very common finding, affecting 3-10% of the general population, mostly women between the fifth and sixth decades of life [1,2]. In almost 80%, they are benign--mostly adenomas--while primary carcinomas are found only in 8% of cases [3]. They can be functional or non-functional, depending on the production of cortisol, aldosterone, androgens, and estrogens [4]. Tumors that produce aldosterone are almost always benign. Glucocorticoid production is seen both in adenomas and carcinomas, but due to the higher frequency of cortical adenomas, most of the time, they are proven to be benign as well, whereas the production of sex hormones is mostly found in carcinomas [5]. Non-functional lesions are mostly detected randomly, and thus they are called incidentalomas. On the other hand, in cases of adrenal cancers, more than 50% of them are functional [4,6]. Benign features include lesions <4 cm and Hounsfield Units (HU) <10 on an unenhanced CT [5,6]. The most common type of benign adrenal lesion is cortical adenoma (80%) [2]. Most of them are small, with a similar appearance to the zona fasciculata [5]. Atypia can be found in some of them, but the number of mitoses is always low (<1 mitosis/50 high-power fields) [5]. As for malignant lesions, the most common--50% of the tumors resected--are metastatic lesions, probably due to the pattern of blood supply and the communication of the adrenal gland with the pulmonary and retroperitoneal lymphatic pathways [7]. The primary site is usually the kidney, lung, colon, rectum, or skin [7]. Following the above, an extremely rare adrenal tumor, with an incidence of 0.5-2 cases per million, is the adrenocortical carcinoma [5]. It is a large tumor with an average size of 10-12 cm, with color indicative of the hormone secretion [1]. Cortisol-producing tumors are usually yellow, whereas sex-hormone-producing adrenocortical carcinomas are mostly red or brown [5]. Half of the cases are non-functional, while cases of aldosterone production are extremely rare [5]. Early diagnosis and appropriate treatment are of great importance due to the metastatic potential [1]. Our report highlights the management of such rare cases, aiming to provide insights into the diagnostic workup, clinical approach, and postoperative follow-up. 

## Case presentation

A 43-year-old male patient presented in December 2025 to our clinic with unregulated hypertension while using a calcium channel blocker and a beta blocker. He reported no other past medical history other than smoking.

The patient underwent a comprehensive biochemical evaluation of adrenal hormonal function. Plasma metanephrine concentration was within the reference range (39 ng/L; reference range, 0-73 ng/L). Similarly, dehydroepiandrosterone sulfate (DHEA-S; 183.4 μg/dL; reference range, 35-570 μg/dL), total testosterone (405.76 ng/dL; reference range, 150-950 ng/dL), free testosterone (10.7 ng/dL; reference range, 4.7-19 ng/dL), and ACTH (48.3 pg/mL; age-adjusted reference range, 7-65 pg/mL) were within normal limits. The baseline 08:00 am serum cortisol concentration was within the age-adjusted reference range (18.2 μg/dL; reference range, 6-25 μg/dL). Following an overnight 1-mg dexamethasone suppression test, the 08:00 am serum cortisol concentration was adequately suppressed to 1.0 μg/dL. Given the presence of poorly controlled arterial hypertension, assessment of the renin-aldosterone axis was also performed while on β-blocker treatment. Plasma aldosterone concentration was 28.1 ng/dL at rest (reference range, 2-26 ng/dL), with a plasma renin activity of 0.15 ng/mL/h at rest (reference range, normal values 0.10-2.3 ng/mL/h), resulting in an elevated aldosterone-to-renin ratio (ARR) of approximately 187 ng/dL per ng/mL/h. Overall, the biochemical evaluation revealed no evidence of catecholamine, androgen, or autonomous cortisol secretion, while the elevated aldosterone-to-renin ratio was considered potentially confounded by concomitant β-adrenergic blockade, which may suppress renin secretion and increase the ARR independently of autonomous aldosterone production.

The patient underwent a CT scan that showed a solid, ovoid 7-cm lesion of the left adrenal gland demonstrating internal attenuation of 30 HU on pre-contrast imaging, increasing to 60 HU following contrast administration and decreasing to 38 HU 15 min later. In the 15-min delayed measurement after contrast administration, the absolute contrast washout was 73% while the relative washout was 37%. These findings are depicted in Figure 1.

**Figure 1:**
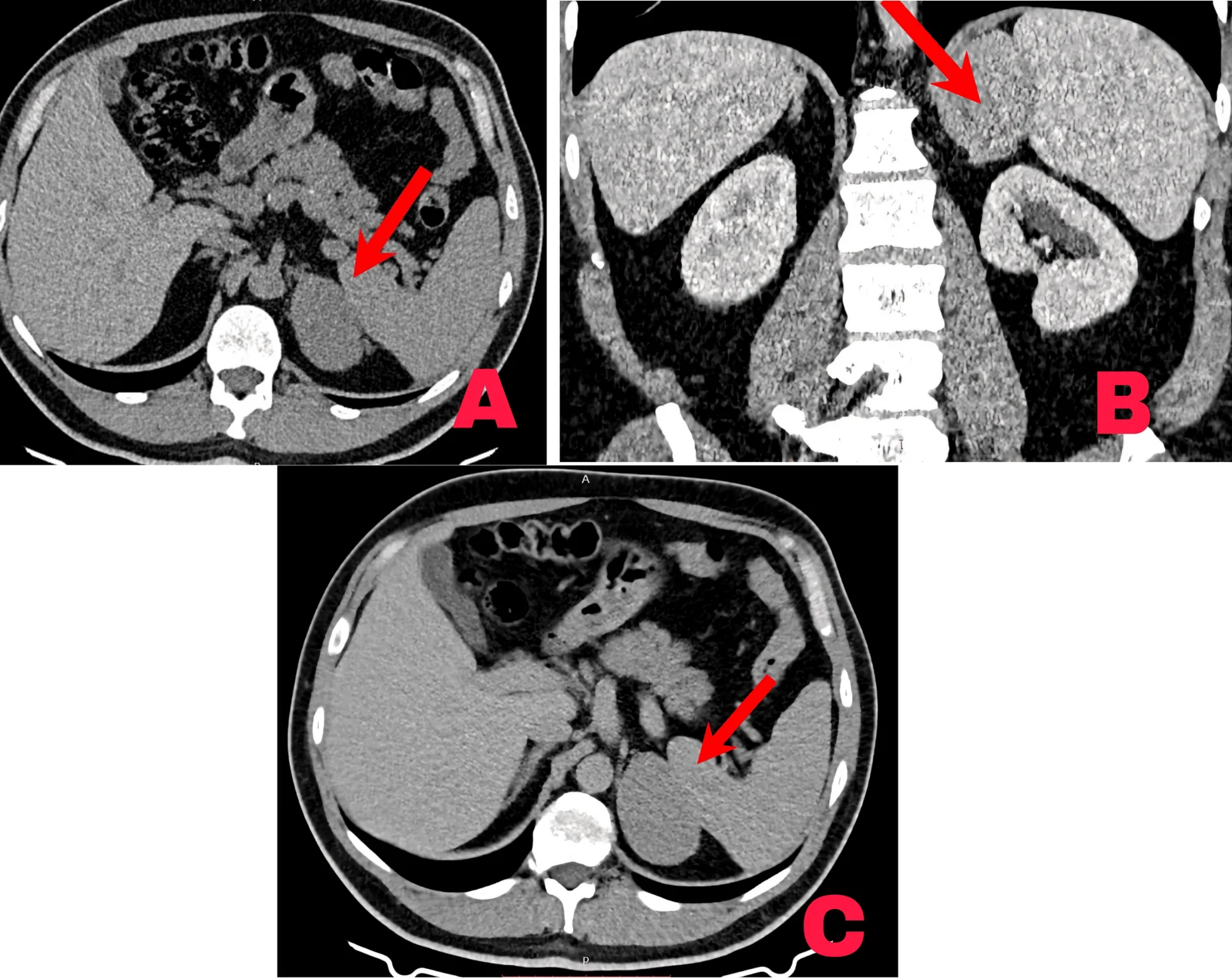
CT scan of the abdomen: depiction of left adrenal lesion (red arrow) An incidental, solid, ovoid 7-cm lesion with internal attenuation of 30 HU on pre-contrast imaging, increasing to 60 HU after contrast administration, and decreasing to 38 HU in a 15-min delayed measurement. A. Axial section pre-contrast; B. Coronal section post-contrast; C. Axial section 15 min after contrast administration HU: Hounsfield Units

For further investigation, the patient was also scheduled for a positron emission tomography-computed tomography (PET-CT), which supported the diagnosis by measuring a 73x45 mm lesion with a maximum standardized uptake value (SUVmax) of 10.5. The scan showed no secondary lesions or pathological lymph nodes (cN0).

He was scheduled in January 2026 for an endoscopic retroperitoneal left adrenalectomy. A posterior retroperitoneoscopic approach was based on the preoperative radiological and clinical assessment of the lesion. At the time of surgery, there was no evidence of local invasion, regional lymphadenopathy, or metastatic disease, and the lesion was considered technically amenable to complete resection without violation of the tumor capsule. If intraoperative findings had demonstrated invasion, inability to obtain adequate margins, or a significant risk of capsular disruption or tumor spillage, the team was prepared for conversion to the open approach. The patient was positioned in the prone jack-knife position for retroperitoneal adrenalectomy; a technique outlined by Walz [8]. An initial incision was made just above the tip of the left twelfth rib, allowing the insertion of a 10 mm trocar. Two additional trocars, measuring 10 mm and 5 mm, were introduced under finger guidance into the space created within Gerota’s fascia following sharp dissection. Carbon dioxide was insufflated and kept at a pressure of 25 mmHg. Once the upper pole of the kidney was identified, the adrenal gland was carefully dissected with ultrasonic shears. The left adrenal vein was then located and ligated. After the adrenal gland was fully removed, the tissue specimen, as seen in Figure 2, was extracted using a retrieval bag.

**Figure 2:**
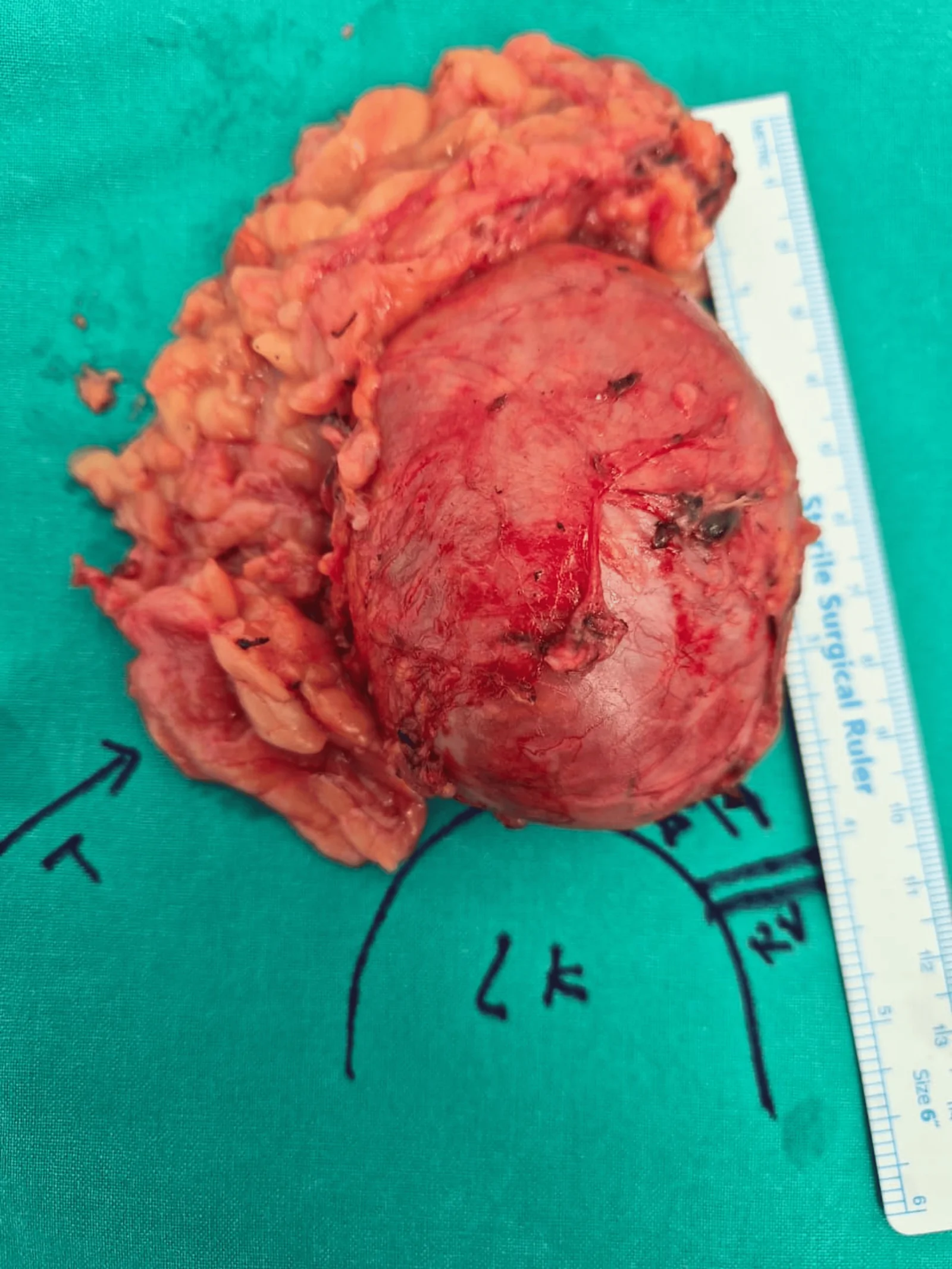
Macroscopic appearance of the left adrenal lesion The specimen demonstrates a large, well-circumscribed adrenal mass measuring approximately 8 cm, with an intact external surface, surrounded by adherent periadrenal tissue.

He recovered quickly and was discharged the next day. His postoperative course was uneventful.

Histological examination revealed an encapsulated oncocytic adrenocortical carcinoma of 75 mm of the adrenal cortex, according to the Lin-Weiss-Bisceglia system [9], as shown in Table 1. Cells were found to be positive for SF1, MART1, Calretinin, CKAE1/AE3--consistent with adrenal origin--while the Ki67 proliferation index was elevated (21%).

**Table 1:** Lin-Weiss-Bisceglia system The diagnosis of malignancy was established based on the presence of one major criterion (11 mitoses/50 HPF) and no minor criteria. No perineural, vascular or sinusoidal invasion was found. HPF: high-power field

Lin-Weiss-Bisceglia system	
Major criteria	
Mitotic rate >5/50 HPF (10 mm²)	Yes - 11 mitoses/50 HPF
Atypical Mitosis	No
Venous Invasion	No
Minor criteria	
Size >10 cm or Weight >200 g	No
Necrosis	No
Capsular Invasion	No
Sinusoidal Invasion	No

After an oncology consultation, the patient is receiving mitotane. He also receives hydrocortisone 50 mg per day. Follow-up imaging studies that were performed in the sixth month showed no evidence of recurrence. The patient is in the eighth month post operation and is being closely monitored by an endocrinologist and an oncologist.

## Discussion

Adrenocortical carcinoma (ACC) is an ultrarare disease with an incidence of 1-2 per million per year and female predominance [1,5]. It appears to be more common in children, while the median age of diagnosis in adults is considered the fifth to sixth decades [1]. Unlike the common epidemiology, our patient is male and 43 years old. They are usually unilateral, large, heavy tumors (approximately 10-12 cm, 300 g) [3,5]. Half of them are functional with a preference for cortisol production, either alone or in combination with a sex steroid [5]. Aldosterone-secreting ACCs are extremely rare [5].

Due to its increased prevalence in childhood, adrenocortical carcinoma is believed to follow a genetic pathway [1]. Although the mechanism is not entirely clear, around 50-80% of children with ACCs have germline TP53 mutations [1], explaining the concurrence with Li-Fraumeni syndrome [4,10,11]. In the adult population, the prevalence of the TP53 mutation is between 4% and 7% [1]. Another commonly seen mutation is that of the 11p15 locus, leading to IGF gene overexpression, thus explaining the association with Beckwith-Wiedemann syndrome [4,11]. High IGF2 expression is also associated with a five times increased risk of recurrence [1]. ACCs have also been related to the multiple endocrine neoplasia type 1 syndrome (MEN1), with several case reports also documenting occurrence in patients with familial adenomatous polyposis (FAP), neurofibromatosis type 1, and Lynch and Werner syndrome [1,12-14]. Non-genetic predisposing factors increasing the risk of adrenocortical carcinoma include cigarette smoking in men and the use of oral contraceptives in women [1,4].

Patients with functional lesions present with symptoms due to the hormonal excess [1]. The most common is hypercortisolism [5], while other commonly heard complaints include male pattern baldness, virilization, menstrual irregularities in women, gynecomastia, and testicular atrophy, seen in patients with sex hormone-producing ACCs [3,4]. Nonfunctional tumors, like in our case, usually cause symptoms such as abdominal pain or fullness, weight loss, night sweats, hematuria, and dyspnea due to their size [1,4]. Paraneoplastic syndromes are uncommon [1].

In cases where adrenocortical carcinoma is suspected, a biochemical evaluation should be made [1,15]. For hypercortisolism, physicians assess the dexamethasone suppression test (DST), the increased measurement of cortisol on a spontaneous 8.00 am blood draw, the midnight salivary cortisol test, and an elevated 24-hour urinary free cortisol [1,4]. DHEA-S and total testosterone should also be measured to confirm a sex-hormone-secreting carcinoma, while screening for aldosterone includes measuring plasma renin activity (PRA) and serum aldosterone levels [1]. As part of the differential diagnosis, assessment of plasma metanephrines, normetanephrines, and 24-hour urine is also important to rule out pheochromocytoma [1,4,15]. Thoraco-abdomino-pelvic (TAP) contrast-enhanced CT or MRI are the imaging options of choice [1,4,15]. ACCs show up with a density of >10 HU (sensitivity 97%, specificity 52%) on CT, as seen in our case [1,15]. On chemical shift MRI, there is a typical appearance of regions of signal loss (<30% of the lesion) due to the intracellular lipid of the ACCs [1,4]. MRI is usually preferred for patients with renal insufficiency to avoid the IV contrast agent [15]. Another imaging technique commonly used is the fluorodeoxyglucose (FDG) PET scan [1]. Except for the disease extension, it also provides us with information favoring malignancy and proving the adrenal origin of the lesion [15]. The use of fine-needle aspiration (FNA) is usually not recommended to avoid the risk of tumor dissemination, and since it cannot distinguish between adrenocortical adenoma and carcinoma, it is of limited significance [4,6,15].

The key feature that distinguishes adrenocortical adenomas from carcinomas, except for metastases, is the presence of invasion [1]. Several scores have been proposed for malignancy, with the most widely used being that of Weiss [1]. The Weiss criteria assign 1 point for each histological feature present, resulting in a scale from 0 to 9, with 3 or more being the score for malignancy [5]. It is observed that all tumors with scores less than 3 pursue a benign course, while 91% of patients with tumor score >3 show recurrence or metastasis, and are later diagnosed as ACCs [5,15]. Histological features that are evaluated are: mitotic activity (>5 per 50 HPFs), presence of atypical mitosis, necrosis, high nuclear grade, venous invasion, sinusoidal invasion, capsular invasion, diffuse growth accounting for more than 1/3 of the tumor, and clear cells comprising 25% of the tumor [3,15]. Other systems proposed to distinguish between benign and malignant adrenal cortical lesions are those of Hough (assessment of both histologic features and clinical symptoms), the reticulin algorithm, and that of Aubert [5,10]. Both the reticulin algorithm and the Aubert algorithm propose modifications to the Weiss scale [1,5,9,10,11,16]. Apart from histological criteria, malignancy can also be confirmed with the Ki-67 proliferation marker (Ki-67 >5% in ACCs), as in our case where Ki-67 was 21% [1,3]. Immunomarkers are used to prove the cortical origin of the lesion as well [1]. The most reliable and distinct markers to confirm steroidogenic tissues are SF-1 (steroidogenic factor 1) and Melan-A [3]. Other biomarkers that are usually used are inhibin alpha, calretinin, and D2-40 [3]. The PAX8 marker can distinguish between ACCs, where it is negative, and metastases from renal cancer, where it is positive, while chromogranin is used to differentiate ACCs from pheochromocytomas (negative in ACCs, positive in pheochromocytoma) [5]. At a percentage >20%, Ki-67 has a strong correlation with worse prognosis [1,3]. Expression levels of DLG7 and PINK1 can predict disease-free survival regardless of tumor grade, while overall survival can be predicted by different levels of BUB1B and PINK1 [1,3].

A few histological variants of ACCs have been described, including the oncocytic, myxoid, and sarcomatoid subtypes, with the latter following a more aggressive course [1].

The European Network for the Study of Adrenal Tumors (ENSAT) staging system defines 4 stages for ACCs [1,17]. Stage 1 (<5 cm) and 2 (>5 cm) are both limited to the adrenal gland, while stage 3 extends into surrounding tissue (locoregional lymph nodes, periadrenal adipose tissue, or adjacent organs). Stage 4 is reserved for patients with metastatic disease, which is observed in 49% of patients at their initial presentation [1,17]. The most common metastatic sites are the lungs (40-80%), liver (40-90%), and bones (5-20%) [1]. Our patient is classified as stage 2-7.5 cm, limited to the adrenal gland.

Regardless of the subtype, the gold-standard treatment for ACCs is adrenalectomy, either open, laparoscopic, or robotic. In cases of ACCs, adrenalectomy should be performed en bloc to avoid tumor distribution due to capsule violation [11,18]. It can be performed transabdominally (TA) or retroperitoneoscopically (PRA), with both techniques presenting equivalent results and recovery [18]. The transabdominal technique is mostly used in large tumors (>6 cm) due to wider working space since PRA makes it difficult for the surgeon to manipulate them [8,18]. The laparoscopic technique is preferred for smaller tumors (<10 cm) [8]. The benefits of this method include lower morbidity, less pain, shorter hospital stays, and decreased overall recovery time [1]. The laparoscopic approach can also be used posteriorly, through the retroperitoneal space, as depicted in our case [19]. For smaller lesions, robotic-assisted adrenalectomy has also been demonstrated [19]. In general, preserving adrenal function is important due to the severe side effects of lifelong steroid replacement, but partial adrenalectomy is not yet recommended for malignancies due to high recurrence rates [19]. Although total adrenalectomy is the treatment of choice for non-metastatic ACCs, the decision for resection in stage 4 patients should be individually addressed [1].

Another undefined area in adrenal surgery is the extent of lymph node dissection [1]. The main lymphatic areas are the renal hilum and the origin of the celiac and mesenteric artery [1]. In 1904, Gregoire was the first to argue that the anterior compartments of the adrenal gland drain towards the latero-aortic lymph nodes, whereas the posterior ones drain at the right and left diaphragmatic pillar and then follow a course toward the thoracic duct and a mediastinal lymph node at the level of the ninth thoracic vertebra [12]. Considering the above as well as the technical difficulty of excising lymph nodes, both near the posterior mediastinum and near the retrocava, the consensus on a lymph node dissection protocol is difficult [11]. A potential option could be adjuvant radiotherapy [12]. While lymph node involvement is reported, lymphadenectomy is not formally performed either because of suspected benign etiology or because of possible complications delaying adjuvant therapy, as it has not been proven to have a clear benefit [1].

In general, chemotherapy and radiotherapy are suggested for advanced and metastasized ACCs [1]. Chemotherapeutic regimens have a 30-50% response rate, when counting stable disease as a response too, due to the expression of the multidrug resistance 1 gene (MDR1) and its protein (Pgp) in adrenal cortical carcinoma cells [1,4,20]. The most promising chemotherapeutic regimen is mitotane [4]. It is also preferred for adjuvant use in patients with increased risk of local recurrence, ENSAT III+IV and Ki67 >10%, and R1 resection [1]. Sometimes, it could be considered even in individual patients with a low risk of recurrence, ENSAT I-II, and Ki67<10% [17]. In our patient, the use of mitotane was decided based on high Ki67 (21%). Plasma concentrations should be >14 mg/L, which causes toxicity, side effects including neurological and GI tract effects, bone marrow suppression, drug-induced adrenal insufficiency, increased liver enzymes, alkaline phosphatase, γ-glutamyl transferase, and thus difficulty to manage [3]. Adjuvant radiotherapy in combination or not with mitotane for stage 3 ACCs or R1, R2 resections is still debated [19]. Due to the poor prognosis of traditional therapies, alternative modes of treatment are being developed [1]. Drugs targeting the IGF-1R system are currently assessed in phase I and II trials [1]. Tyrosine kinase inhibitors and immune checkpoint inhibitors have also been tested in phase II trials, but they do not show greater effectiveness than mitotane [17]. However, it is important to note that therapy with ipilimumab and nivolumab did show a response in tumors with microsatellite instability (MSI) [19]. In palliative cases, several case reports have demonstrated that radiotherapy can provide symptom relief [1]. Alternative options include ketoconazole and metyrapone for control of glucocorticoid overproduction, mifepristone for the same purpose, and spironolactone to address both androgen and mineralocorticoid effects [1].

## Conclusions

Adrenocortical carcinomas represent rare lesions with high metastatic potential, related to genetic pathways. Diagnosis is based on a hormonal workup and radiological findings preoperatively, while postoperatively, the histological examination confirms the findings. Surgical excision remains the gold standard treatment depending on the stage and size of the tumor.

Nevertheless, there is a great need for larger studies given the persistent gaps in the literature regarding therapeutic options to provide physicians with a framework and establish comprehensive clinical guidelines.
